# Increased prevalence of eating disorders in Japan since the start of the COVID-19 pandemic

**DOI:** 10.1007/s40519-021-01339-6

**Published:** 2021-12-02

**Authors:** Ken Kurisu, Mikiko Matsuoka, Kaoruko Sato, Asako Hattori, Yukari Yamanaka, Nobuhiro Nohara, Makoto Otani, Kazuhiro Yoshiuchi

**Affiliations:** Department of Stress Sciences and Psychosomatic Medicine, Graduate School of Medicine, The University of Tokyo, 7-3-1, Hongo, Bunkyo-ku, Tokyo, 113-8655 Japan

**Keywords:** Eating disorders, COVID-19, Confinement, Exercise, Fatness phobia, Japan

## Abstract

**Purpose:**

The study aimed to investigate the effect of the COVID-19 pandemic on the prevalence of eating disorders in Japan.

**Methods:**

We retrospectively reviewed the medical records of new patients with eating disorders who visited an outpatient eating disorders clinic of a single university hospital in Tokyo, Japan, from April 2020 to March 2021 (FY2020) and April 2019 to March 2020 (FY2019). We determined whether the onset or course in each patient in FY2020 was associated with the COVID-19 pandemic and classified COVID-19-associated medical histories into the following categories: (1) fatness phobia, (2) acceleration of dieting, (3) family relationships, (4) social factors, and (5) mood change. We performed the Kolmogorov–Smirnov test to compare the cumulative distribution of disease onset by month in FY2020 and FY2019.

**Results:**

We reviewed the records of 112 and 77 patients with eating disorders in FY2020 and FY2019, respectively. The onset or course of 35 patients (31.3%) in FY2020 was associated with the COVID-19 pandemic. We classified 14 patients to fatness phobia category, 11 to acceleration of dieting, 4 to family relationships, 2 to social factors, and 4 to mood change. No COVID-19-associated cases were associated with fear of contracting the disease. The cumulative distribution of disease onset differed significantly in FY2020 and FY2019 (*D* = 0.248; *P* = 0.007).

**Conclusion:**

This chart review suggests that the COVID-19 pandemic may increase the prevalence of eating disorders.

**Level of evidence:**

III, cohort study.

**Supplementary Information:**

The online version contains supplementary material available at 10.1007/s40519-021-01339-6.

## Introduction

The COVID-19 pandemic reportedly worsens symptoms in patients previously diagnosed with eating disorders through several factors, including deteriorated family relationships, lifestyle changes, mood changes, fear of contagion, and lack of social support [1–4]. In the general population, eating behaviors and body image have been reportedly associated with several factors related to the COVID-19 pandemic, such as fear, anxiety, and intolerance of uncertainty [5, 6]. Furthermore, a report suggested an increase in patients with eating disorders since the COVID-19 pandemic [7]. Although these reports, which are from Europe, the Middle East, New Zealand, and the United States, suggest that the COVID-19 pandemic may increase the prevalence of eating disorders, there have been few reports showing such an increase in eating disorders in the Asian region, including Japan. However, since the state of emergency that the Japanese government declared in April 2020, we have encountered multiple patients with eating disorders whose onset was associated with the COVID-19 pandemic at our hospital in Tokyo, Japan. For example, we experienced patients who had no dieting behavior and had been normal weight but started a pathological diet after gaining weight due to decreased physical activity that occurred after shifting to teleworking or school closure recommended by the Japanese government. Such cases led us to hypothesize that the COVID-19 pandemic increases the prevalence of eating disorders in Japan. This study aimed to investigate the impact of the COVID-19 pandemic on the prevalence of eating disorders.

## Materials and methods

### Ethics approval

This retrospective study was conducted at a single university hospital. This study was approved by the Institutional Review Board of the University of Tokyo Hospital (approval number: 3375). The requirement for informed consent was waived because the study was retrospective. We posted the study on the website of the Department of Psychosomatic Medicine at the University of Tokyo Hospital so that patients could reject the utilization of their data according to the guideline by the Ministry of Health, Labor, and Welfare. No patient requested data removal from the analysis.

### Medical chart review

We reviewed the medical records of new patients with eating disorders who visited the Department of Psychosomatic Medicine at the University of Tokyo Hospital outpatient clinic from April 2020 to March 2021 (FY2020) and April 2019 to March 2020 (FY2019). The Japanese fiscal year starts in April. Social changes, including the declaration of the state of emergency that recommended shifting to teleworking, reducing business hours, and school closures, were concentrated around April 2020 in Japan [8]. Thus, these periods could represent the pre-pandemic (FY2019) and post-pandemic (FY2020) periods, which seemed suitable for the study. We assumed that there was no noteworthy difference between FY2020 and FY2019 in the healthcare system or in social conditions surrounding patients with eating disorders except for the COVID-19 pandemic, and we thus compared these two periods. The data were collected from the electronic medical records of the hospital. The chart review was performed by two or more authors.

We collected data on age, sex, diagnosis [anorexia nervosa restricting-type (ANR), anorexia nervosa binge-purging type (ANBP), bulimia nervosa (BN), binge eating disorder (BED), avoidant/restrictive food intake disorder (ARFID), and others or undetermined] according to the Diagnostic and Statistical Manual of Mental Disorders 5th edition [9], and time of disease onset. The diagnoses and time of disease onset were determined by psychosomatic physicians specialized in eating disorders. In usual clinical practices, we determine the time of disease onset comprehensibly considering symptom onset, changes in body weight, history of diagnosis or treatment at other hospitals, and information from families. Further, we also check psychological and social factors around disease onset and change in symptoms. This information is documented in the electronic medical record.

### Classification of medical histories

We also confirmed whether the onset or course of each patient in FY2020 was associated with the COVID-19 pandemic. For patients in FY2020 who had such an association, their medical histories were classified based on expert opinion. Specifically, three psychosomatic physicians (K.K., K.S., and A.H.) independently classified each patient based on their medical history, and subsequently, the classification was finalized through discussion among all the authors.

We considered the following categories of medical history associated with the COVID-19 pandemic: (1) fatness phobia, (2) acceleration of dieting, (3) family relationships, (4) social factors, and (5) mood change. The detail of the definition is shown in Supplementary Table 1. In summary, the fatness phobia category includes patients who started to have or deteriorate fatness phobia after a change in lifestyle due to the COVID-19 pandemic. The acceleration of dieting category includes patients who had dieting behavior or intention within normal limits and then accelerated it after the pandemic. The family relationships category includes patients who felt family relationships as a stressor after the pandemic and then started to have or deteriorate symptoms of eating disorders. The social factors category includes patients who felt social factors as a stressor after the pandemic and then started to have or deteriorate symptoms of eating disorders. Finally, the mood change category includes patients who experienced significant mood changes after the pandemic and then started to have or deteriorate symptoms of eating disorders.

### Statistical analysis

To compare the means of continuous variables (age) between patients in FY2020 and those in FY2019, we applied a *t* test (Student’s or Welch’s) or the Mann–Whitney *U*-test after examining variance homogeneity using the *F*-test and normality using the Kolmogorov–Smirnov test. We used the Chi-square test or Fisher’s exact test to compare the proportions of categorical variables (sex and diagnosis) between the groups.

To quantitatively validate our hypothesis, we compared the cumulative distribution function for disease onset by month between the groups using the Kolmogorov–Smirnov test [10]. In this analysis, for patients in FY-*X* (*X* = 2019 or 2020), the month of onset in January *X*–December *X* was coded as 1–12, January *X* + 1–March *X* + 1 as 13–15, and before January *X* as 0. We also performed this analysis for subgroups according to diagnosis (ANR, ANBP, BN, and BED).

All statistical analyses were conducted using R version 4.1.1 (R Foundation for Statistical Computing, Vienna, Austria, 2021). A *P* value < 0.05 was considered statistically significant.

## Results

We reviewed 112 patients in FY2020 and 77 patients in FY2019 (Table 1). There were no significant differences in age, sex, or diagnosis between the groups.

**Table 1:** Patients’ characteristics

	FY 2020 (*N* = 112)	FY 2019 (*N* = 77)	*P* value
*Age*			
Mean (SD)	23.29 (9.81)	25.01 (11.07)	0.32^a^
Median (Range)	19.5 (12–55)	20 (12–58)	
*Sex, n (%)*			> 0.99^b^
Male	4 (3.6)	3 (3.9)	
Female	108 (96.4)	74 (96.1)	
*Diagnosis, n (%)*			0.32^b^
ANR	57 (50.9)	32 (41.6)	
ANBP	28 (25.0)	26 (33.8)	
BN	13 (11.6)	8 (10.4)	
BED	6 (5.4)	8 (10.4)	
ARFID	2 (1.8)	2 (2.6)	
Other or undetermined	6 (5.4)	1 (1.3)	

FY2020 and FY2019 correspond to April 2020–March 2021 and April 2019–March 2020, respectively

^a^Mann–Whitney *U* test

^b^Fisher’s exact test

SD, standard deviation; ANBP, anorexia nervosa binge-purging type; ANR, anorexia nervosa restriction type; ARFID, avoidant/restrictive food intake disorder; BED, binge eating disorders; BN, bulimia nervosa

Thirty-five patients in FY2020 (31.3%), including 25 patients with ANR, 3 with ANBP, 4 with BN, 1 with BED, and 2 with other or undetermined eating disorders, were classified as having an onset or course associated with the COVID-19 pandemic. We classified 14 patients to fatness phobia category, 11 to acceleration of dieting, 4 to family relationships, 2 to social factors, and 4 to mood change. The details of the medical history are shown in Supplementary Table 2. No patient belonged to multiple categories.

**Fig. 1:**
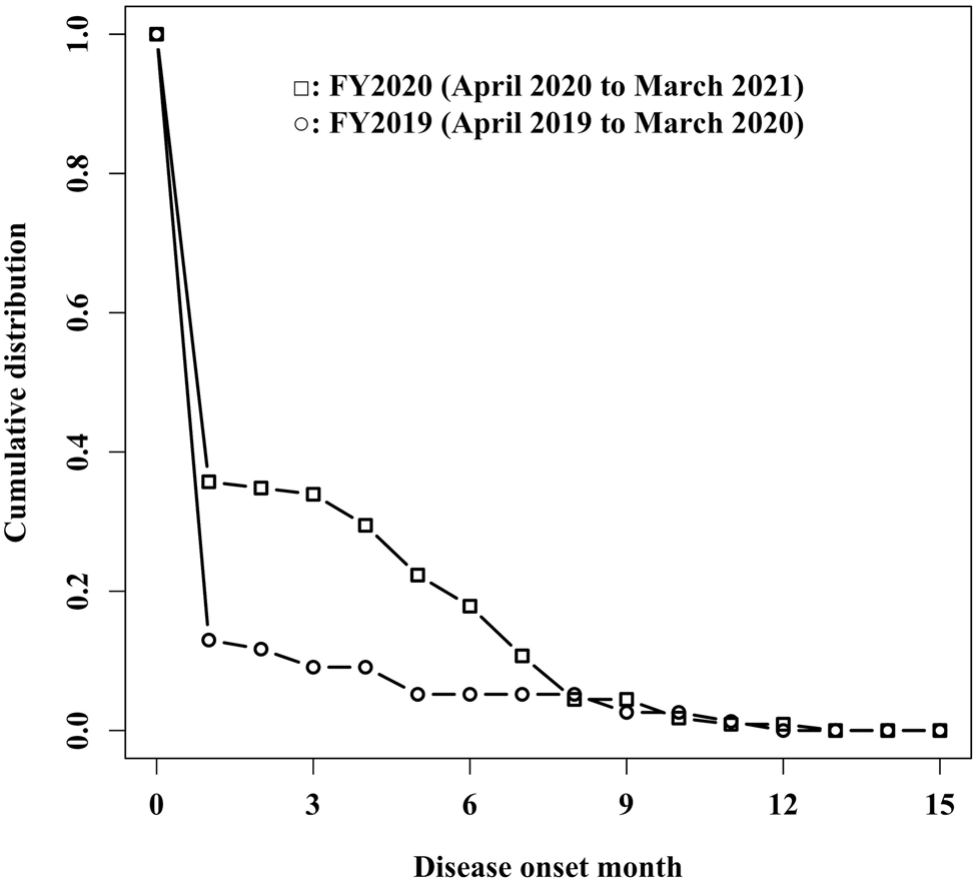
Cumulative distribution function of months of disease onset for new patients who visited the outpatient clinic in each fiscal year. For patients in FY[*X*] (*X* = 2019, 2020), the month of onset in January *X*–December *X* was coded as 1–12, January *X* + 1–March *X* + 1 as 13–15, and before January *X* as 0

Figure 1 shows the cumulative distribution of months of disease onset for patients in each period. The Kolmogorov–Smirnov test showed that the cumulative distribution of disease onset was significantly different between the groups (*D* = 0.248; *P* = 0.007). The results of the subgroup analyses are shown in Supplementary Table 3. There was significant difference only in the group of ANR (*D* = 0.331; *P* = 0.022).

## Discussion

We reviewed the medical charts of new patients with eating disorders who visited our hospital. The onset or course of approximately one-third of patients with eating disorders in FY2020 appeared to be associated with the COVID-19 pandemic. This supports our hypothesis that the COVID-19 pandemic increased the prevalence of eating disorders.

Furthermore, the distribution of disease onset by month differed significantly between FY2020 and FY2019, with other variables showing no significant differences. The difference in cumulative distribution function is large between the month codes from 0 to 8 but is almost identical after the month code of 9. This might be explained by short periods for patients whose onset was after August to visit hospitals within the fiscal year. The result quantitatively suggests that the onset trigger may have differed by year, supporting our hypothesis that the prevalence of eating disorders increased in association with the COVID-19 pandemic.

These results are consistent with those of previous reports from other countries. First, the COVID-19 pandemic worsens symptoms in patients already diagnosed with eating disorders [1–4]. Second, the pandemic also influences eating behavior in the general population [5, 6]. Finally, the number of adults with eating disorders has reportedly increased [7].

Of the patients with the onset or course related to the COVID-19 pandemic, the most common were those with ANR. Further, the subgroup analysis showed a significant difference only in the group of ANR, although the other subgroups had a relatively small sample size. These results suggest that the implications of this study apply mainly to patients with ANR. This may be plausible because we observed short periods after the disease onset, and thus these ANR patients may be before they transition to ANBP or other disease types.

The several categories of medical histories associated with the COVID-19 pandemic determined in this study, including fatness phobia, acceleration of dieting, family relationships, and mood disturbance, are consistent with the factors influencing eating disorder pathology revealed in previous studies [1–6]. In contrast to previous studies [4, 5], this study identified no patients who reported fear of SARS-CoV-2 infection as the cause of their disease. The relatively low number of deaths due to COVID-19 in Japan in 2020 might explain this difference [8]. The factors through which the COVID-19 pandemic influences eating disorders may vary depending on the pandemic situation in each country.

The results of this study indicate that clinicians should be aware of the impact of the COVID-19 pandemic on the onset or course of eating disorders when taking a medical history and assessing the condition of patients with eating disorders. However, it remains unclear whether the prognosis of eating disorders differs according to whether they are associated with the COVID-19 pandemic. Appropriate psychoeducation may lead to a better course of treatment for patients whose onset was triggered by the pandemic. For example, a study can be conducted to provide psychoeducation through social media, which would also have a preventive effect. Further investigations are warranted to determine the clinical implications of this study’s results.

### Strengths and limitations

A strength of this study is that, to our knowledge, it is the first study conducted in Japan that provides evidence of an increase in eating disorders triggered by the COVID-19 pandemic. There have been several reports of an association between the pandemic and eating disorders in other regions. Our study provides supporting evidence that pandemic-associated eating disorders are a worldwide issue.

This study had several limitations. First, the study was conducted in a single center, so the findings might not be generalizable. Second, the significant difference in the cumulative distribution of months of disease onset might be attributable to factors other than the COVID-19 pandemic. Thus, larger cohort studies are required for quantitative validation. Third, the assumption that there was no noteworthy difference between FY2020 and FY2019 in the healthcare system or social conditions surrounding patients with eating disorders might be incorrect. Fourth, the data obtained from the electronic medical records can be influenced by the subjectivity of psychosomatic physicians in charge and might have missing descriptions. Fifth, we cannot determine whether the patients who developed eating disorders after the start of the COVID-19 pandemic would have developed eating disorders in the absence of the pandemic. Sixth, this study’s participants were mostly female; thus, the results have no generalizability to male patients. Seventh, the definition for each category of medical histories was determined retrospectively and was subjective. Further research, such as a study using content analysis, will be required to refine the classification. Finally, the study did not follow-up patients with eating disorders to determine the prognosis and outcome.

## Conclusion

We identified many patients with eating disorders whose onset or course was associated with the COVID-19 pandemic, mainly through fatness phobia induced by changes in lifestyle. This result suggests that the COVID-19 pandemic may increase the prevalence of eating disorders in the Japanese population. Further investigations are warranted to validate the findings.

### What is already known on this subject?

There have been reports from countries outside the Asian region that the COVID-19 pandemic has worsened the symptoms of patients with eating disorders and increased the incidence of eating disorders.

### What this study adds?

This study suggests that the COVID-19 pandemic may have led to an increased prevalence of eating disorders in Japan.

## Supplementary Information

Below is the link to the electronic supplementary material.

Supplementary file1 (DOCX 28 kb)

## Data Availability

The datasets are available from the corresponding author on reasonable request.

## Code availability

The source codes are available from the corresponding author on reasonable request.
