# Supplementary material for: Increased prevalence of eating disorders in Japan since the start of the COVID-19 pandemic
Source: Eat Weight Disord. 2021 Dec 2;27(6):2251–5. doi: 10.1007/s40519-021-01339-6 (PMC8638639; doi:10.1007/s40519-021-01339-6)
Supplement: Supplementary file 1 — Supplementary file1 (DOCX 28 kb) [file 40519_2021_1339_MOESM1_ESM.docx]

**Supplementary Table 1. Definition of categories for medical history associated with the COVID-19 pandemic.**

| Category | Definition |
| --- | --- |
| Fatness phobia | ・Fatness phobia, which did not exist or was stable if it existed before, appeared or worsened after the change in lifestyle caused by the COVID-19 pandemic.  ・The interval between the change in lifestyle and the appearance or worsening of fatness phobia was almost within a month. |
| Acceleration of dieting | ・Dieting behavior or intention existed before the COVID-19 pandemic but was within the normal level.  ・The change in lifestyle caused by the COVID-19 pandemic accelerated the behavior.  ・The interval between the change in lifestyle and the acceleration in the behavior was almost within a month. |
| Family relationships | ・They felt family relationships as a significant stressor, which was caused by changes in lifestyle due to the COVID-19 pandemic.  ・The onset or exacerbation of eating disorder symptoms from the appearance of the stressor was almost within a month. |
| Social factors | ・Social environment, such as work and school life, changed due to the COVID-19 pandemic, and they felt this was a significant stressor.  ・Changes in the social environment do not include those in family relationships.  ・The onset or exacerbation of eating disorder symptoms from the appearance of the stressor was almost within a month. |
| Mood change | ・They had mood changes, such as depression and anxiety, due to the COVID-19 pandemic.  ・The onset or exacerbation of eating disorder symptoms from the mood change was almost within a month. |

**Supplementary Table 2. Number of patients belonging to each category and specific association between their courses and the COVID-19 pandemic.**

| Category | N | Association |
| --- | --- | --- |
| Fatness phobia | 10 | Decreased physical activity induced fatness phobia as a reaction. |
|  | 3 | Increased food intake induced fatness phobia as a reaction. |
|  | 1 | After resuming school, fatness phobia appeared. |
| Acceleration of dieting | 9 | Spending more time at home accelerated dieting behavior. |
|  | 1 | Spending more time at home increased binge-eating. |
|  | 1 | Spending more time at home increased self-induced vomiting. |
| Family relationships | 2 | Household workload increased, and then food intake decreased. |
|  | 1 | Increased contact with her mother, with whom she was not on good terms, worsened her anorexia. |
|  | 1 | Family relationships deteriorated, and then binge-eating started. |
| Social factors | 1 | Dissatisfaction with school closure induced self-induced vomiting. |
|  | 1 | After resuming school, she felt it as a stressor, and then food intake decreased. |
| Mood change | 2 | Depressive mood by confinement induced anorexia. |
|  | 1 | Anxiety about vomiting increased after decrease in communication, and then food intake decreased. |
|  | 1 | After resuming school, depressive mood started, and then food intake decreased. |

^a^No patient belonged to multiple categories.

**Supplementary Table 3. Kolmogorov-Smirnov tests for subgroups according to diagnoses**

|  | N (FY2020) | N (FY2019) | D-value | P-value |
| --- | --- | --- | --- | --- |
| All diagnoses | 112 | 77 | 0.248 | 0.007 |
| ANR | 57 | 32 | 0.331 | 0.022 |
| ANBP | 28 | 26 | 0.179 | 0.78 |
| BN | 13 | 8 | 0.337 | 0.63 |
| BED | 6 | 8 | 0.000 | >0.99 |

ANBP, anorexia nervosa binge-purging type; ANR, anorexia nervosa restriction type; BED, binge eating disorders; BN, bulimia nervosa. FY2020 and FY2019 correspond to April 2020–March 2021 and April 2019–March 2020, respectively.
